# Supplementary material for: Identification of photoperiod-induced specific miRNAs in the adrenal glands of Sunite sheep (Ovis aries)
Source: Front Vet Sci. 2022 Jul 22;9:888207. doi: 10.3389/fvets.2022.888207 (PMC9354845; doi:10.3389/fvets.2022.888207)
Supplement: Supplementary file 1 [file Data_Sheet_1.ZIP › Supplementary file/miRDeep2 file.zip!/miRDeep2 file/Novel_Structure/SPLP42A3/Novel_500.pdf]

Provisional ID	:	NC_019467.2_19910
Score total	:	1.4
Score for star read(s)	:	-1.3
Score for read counts	:	0
Score for mfe	:	1.1
Score for randfold	:	1.6
Score for cons. seed	:	
Total read count	:	16
Mature read count	:	16
Loop read count	:	0
Star read count	:	0

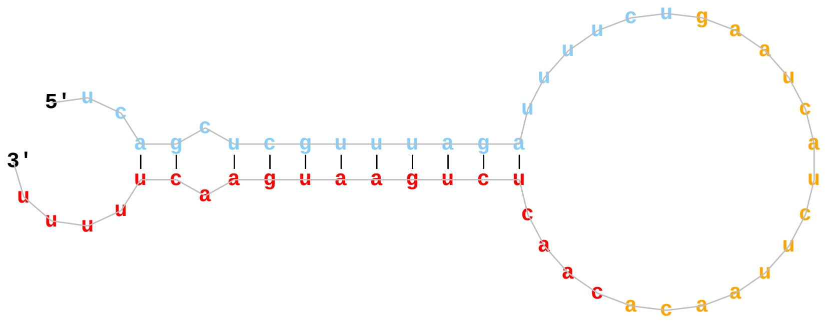

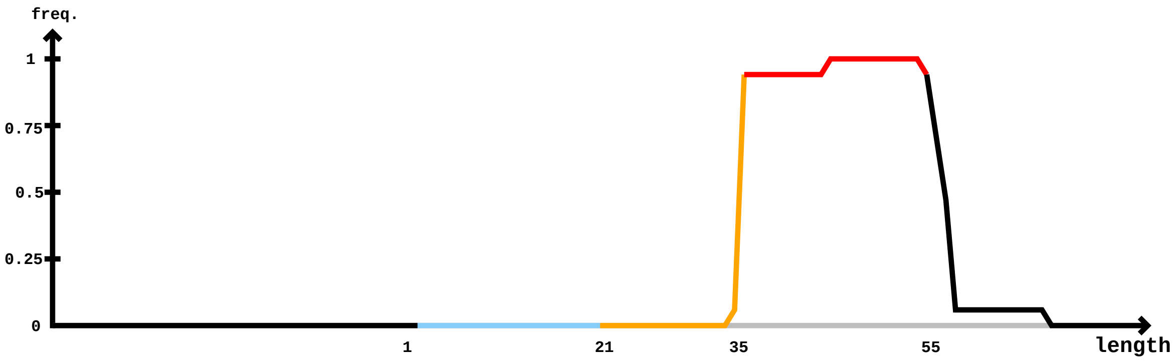

Star Mature

```
5'- uucugagcuugguauuagugauauuggcuuggcuaaucagcucguuuagauuuucugaaucaucuuaacacaacucugaaugaacuuuuuggccagcccaaauacaagcuca   -3'    exp
    ...((((((((.((((..((.....((((.((((((..((.(((((((((........................))))))))).))..))))))))))))))))))))))))    reads    mm    sample
    .....................................................................acaacucugaaugaacuuuuu......................    1        0     seq
    ......................................................................Aaacucugaaugaacuuuu.......................    1        1     seq
    ......................................................................Aaacucugaaugaacuuuuu......................    6        1     seq
    ......................................................................caacucugaaugaacuuuuu......................    1        0     seq
    ......................................................................Aaacucugaaugaacuuuuugg....................    5        1     seq
    ......................................................................caacucugaaugaacuuuuugg....................    2        0     seq
    ...............................................................................augaacuuuuuggccaAcccaaa..........    1        1     seq
```
